# Dual RNA-seq reveals the complement protein C3-mediated host-pathogen interaction in the brain abscess caused by *Staphylococcus aureus*

**DOI:** 10.1128/msystems.01540-24

**Published:** 2025-02-26

**Authors:** Qiyuan Jin, Yaxuan Zhai, Rui Qiang, Xin Ma, Chenhao Zhao, Jinqi Zhong, Jijie Li, Qi Chen, Mingxiao Han, Hong Du, Qifei Cong, Haifang Zhang

**Affiliations:** 1 Department of Clinical Laboratory, The Second Affiliated Hospital of Soochow University, Suzhou, China; 2 MOE Key Laboratory of Geriatric Diseases and Immunology, Soochow University, Suzhou, Jiangsu, China; 3 Institute of Neuroscience, Soochow University, Suzhou, China; 4 Department of Neurology and Clinical Research Center of Neurological Disease, The Second Affiliated Hospital of Soochow University, Suzhou, China; 5 Department of Nephrology, The Second Affiliated Hospital of Soochow University, Suzhou, China; Zhejiang University School of Medicine, Hangzhou, Zhejiang, China

**Keywords:** dual RNA-seq, C3, pathogen-host interaction, brain abscess, *Staphylococcus aureus*

## Abstract

This study aimed to elucidate the complement protein C3-mediated host-pathogen interaction in the brain abscess caused by *Staphylococcus aureus* infection. Dual RNA-seq was employed to analyze the transcriptomic differences between C3 deficiency and wild-type mice of *S. aureus-*induced brain abscess model, and then we investigated the potential regulatory pathways of *S. aureus-*host interaction mediated by C3 and *S. aureus* genes associated with the pathogenesis of brain abscess. Finally, C3 deficient-mice and *hla* mutants of *S. aureus* were used to verify the specific pathogen-host interaction. In the *S. aureus-*induced brain abscess mouse model, the transcriptomic analysis revealed significant changes in bacterial virulence factors, such as hemolysin. Based on these data, we predicted a regulatory network formed by genes like *hrcA* and *dnaK*, which represent a possible regulation mechanism of *S. aureus* responding to the host. Furthermore, we identified that *hla* was the C3 response gene in *S. aureus*. From the host perspective, we observed that the absence of C3 significantly impacted the host’s inflammatory response, primarily by altering the gene expression of several key immune and inflammatory pathways. These findings suggest that C3 deficiency may impair the host’s ability to recognize and respond to external pathogens. To the best of our knowledge, this study proposed that *S. aureus* may affect host immune response through C3, and C3 plays a critical role in regulating inflammation and immune signaling pathways in the brain abscess caused by *S. aureus* infection.

**IMPORTANCE:**

In this work, we employed immunofluorescence and Western blot analysis to reveal a significant upregulation of microglia-derived C3 in the brain abscess mice model caused by *S. aureus* infection. By integrating the individual RNA sequencing data of *S. aureus* and the dual RNA-seq data of *S. aureus* infection brain abscess mice model, the potential regulatory pathways between *S. aureus* and host were identified, and host C3 not only affects the immune response but also mediates the regulation network of *S. aureus*. This study provided the potential novel targets for therapeutic strategies in mitigating the effects of *S. aureus* infections and improving treatment outcomes.

## INTRODUCTION

Brain abscesses are typically caused by pathogens such as bacteria, fungi, and parasites that infect the central nervous system (CNS), leading to brain tissue inflammation and abscess formation, with common pathogens including *Staphylococcus*, *Streptococcus*, and *Escherichia coli* (1) Recent studies indicated that the incidence rate of brain abscess is approximately 0.76 per 100,000 person-years (2). Despite the development of new antimicrobial treatments and advanced diagnostic technologies, brain abscess remains a serious threat to human health due to its severity, rapid progression, and high mortality rate (3).

A recent meta-analysis revealed that 18% (1,076 of 5,894) of culture-positive brain abscess were due to *Staphylococcus* infections (3). Surgical site infections occur in 1%–3% of craniotomy surgeries, half of which are caused by *Staphylococcus aureus* (SA) (4, 5). A recent study analyzed the main pathogens causing brain abscesses over the past decade and found that *S. aureus* ranks as the top cause of brain abscesses among all gram-positive bacterial infections (6). The emergence of methicillin-resistant *S. aureus* and vancomycin-intermediate *S. aureus* has exacerbated the problem of bacterial antibiotic resistance, which can lead to treatment failure in *S. aureus* infections (7).

Microglia are the resident macrophages in the CNS responsible for phagocytosis of cellular debris, production of inflammatory responses, and modulation of synaptic plasticity (8, 9). During viral brain infection or stroke, microglia eliminate damaged synapses to maintain brain stability (10–12). In brain abscess caused by *S. aureus*, microglia respond rapidly through toll-like receptors to recognize bacterial components like peptidoglycan (13, 14). The interaction between microglia and the classical complement system in neurological diseases has been attributed to neuroinflammation and complement-mediated phagocytosis by microglia (15, 16). Recent studies have indicated that classical complement proteins C3 and C1q are involved in synaptic pruning during neuronal development (17, 18) and synaptic loss in neurodegenerative processes (11, 12). Upon infection, complement proteins are activated and form a cascade that leads to the opsonization of pathogens, recruitment of inflammatory cells, and direct lysis of bacteria (19, 20). In the process of *S. aureus* infection, microglia utilize complement receptor to mediate phagocytosis of complement-opsonized bacteria (21). Moreover, it was reported that microglia can generate C3 in various neurodegenerative disorders (22–24).

Dual RNA-Seq is an advanced transcriptomics technology that can simultaneously capture and analyze the gene expression changes of pathogens and host cells during infection. Through this technology, researchers can fully reveal the interaction mechanism between the host and pathogens and explore the unexamined changes in gene expression during infection (25–27).

In this study, we explored the effects of C3 on both the host and the bacteria during brain *S. aureus* infection using dual RNA-seq and found that a multitude of regulatory pathways in both the host and the pathogen are involved in *S. aureus*-host interaction mediated by C3 during the process of CNS infection. Specifically, *S. aureus* forms a gene regulatory network, including genes such as *hrcA* and *dnaK*, which help the bacterium adapt within the host. In the host, over 600 genes are differentially expressed following *S. aureus* infection, among which multiple immune, inflammatory, and complement-related pathways are enriched. Moreover, our data confirm that C3 plays a critical role in amplifying the host’s inflammatory response to *S. aureus* infection. These findings highlight the mechanism in brain infections following *S. aureus* and provide potential therapeutic targets and strategies for the treatment of subsequent brain abscess.

## RESULTS

### Microglia-derived C3 is upregulated during *S. aureus* infection

**Fig 1:**
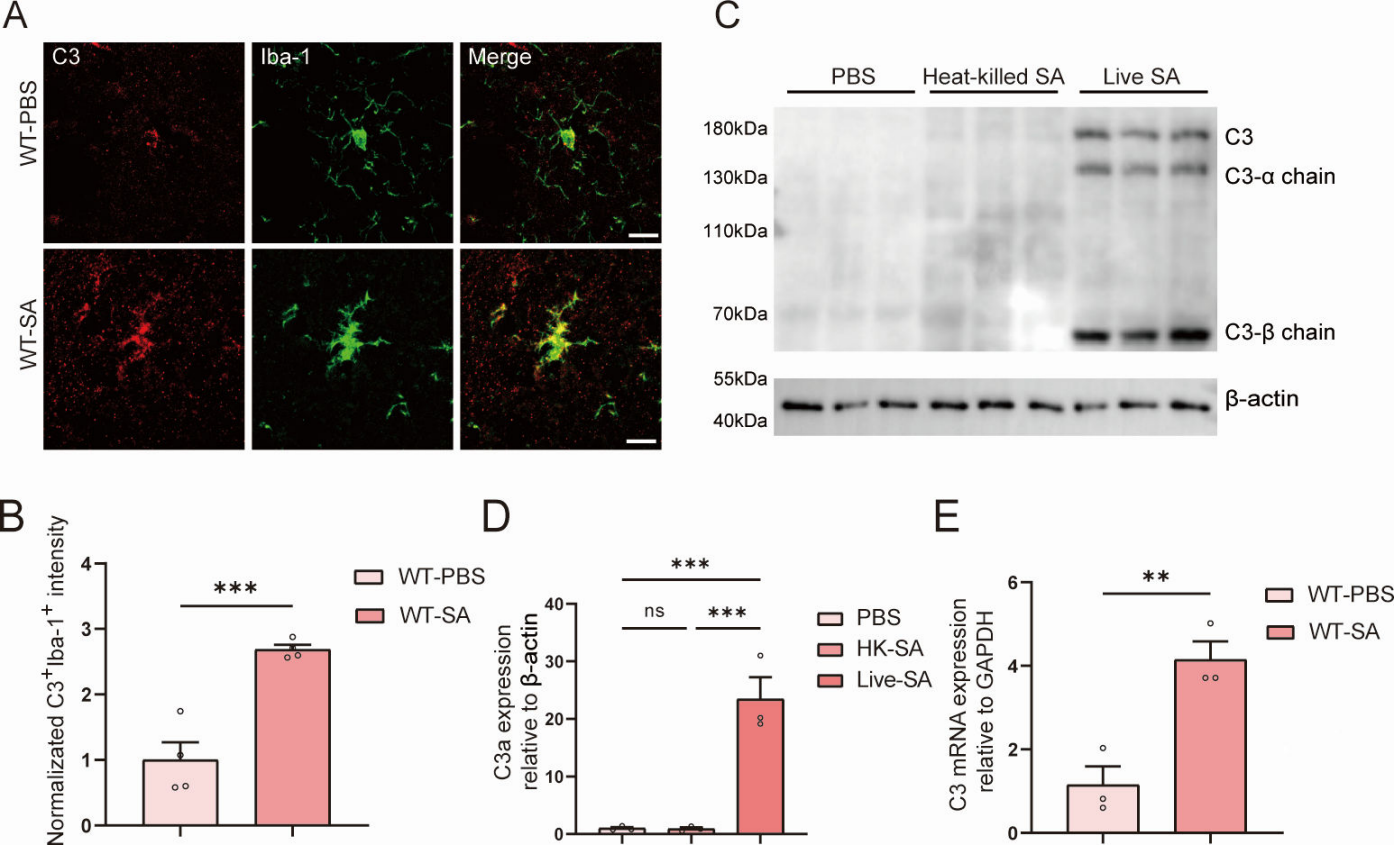
Microglia-derived C3 is upregulated during *S. aureus* infection. (**A**) Immunostaining of C3 (red) and Iba-1 (green) in the striatum from C57BL/6 mice infected with *S. aureus* (wild type [WT]-SA) and treated with phosphate-buffered saline (PBS) (WT-PBS) as a control. Scale bar = 15 µm. (**B**) Quantification of colocalized C3 and Iba-1. (**C**) Immunoblotting analysis of the expression of complement protein C3 α-chain. (**D**) Quantification of the expression of complement protein C3 α-chain. (B and D) Respective quantification of panels A and C. *n* = 3 mice per group. (**E**) C3 mRNA expression levels were determined by real-time qPCR (*n* = 3 mice for each group; for C3, *P* = 0.0085). All data are presented as the mean ± SEM. Unpaired *t*-test was used. **P < 0.01, ***P < 0.001, versus WT-PBS group.

In order to demonstrate that microglia produce C3 after being infected with *S. aureus*, mouse brain sections were immunostained for ionized calcium-binding adaptor molecule 1 (Iba-1, a macrophage/microglial marker) and complement protein C3 (Fig. 1A and B). We found that colocalization between microglia and C3 increased after infection (Fig. 1A and B). This result indicated that microglia are capable of upregulating C3 expression in response to external *S. aureus* stimuli. Western blot experiments further confirmed that infection with live *S. aureus* stimuli led to the upregulation of C3 in BV2 cells, whereas heat-killed *S. aureus* did not have any effects (Fig. 1C and D). Real-time quantitative PCR (RT-qPCR) experiments also revealed that C3 mRNA was upregulated in BV2 cells following *S. aureus* infection (Fig. 1E). Therefore, our results suggest that microglia are capable of secreting C3 during the process of *S. aureus* invasion into the central CNS.

### C3 mediates the inflammatory response of microglia

**Fig 2:**
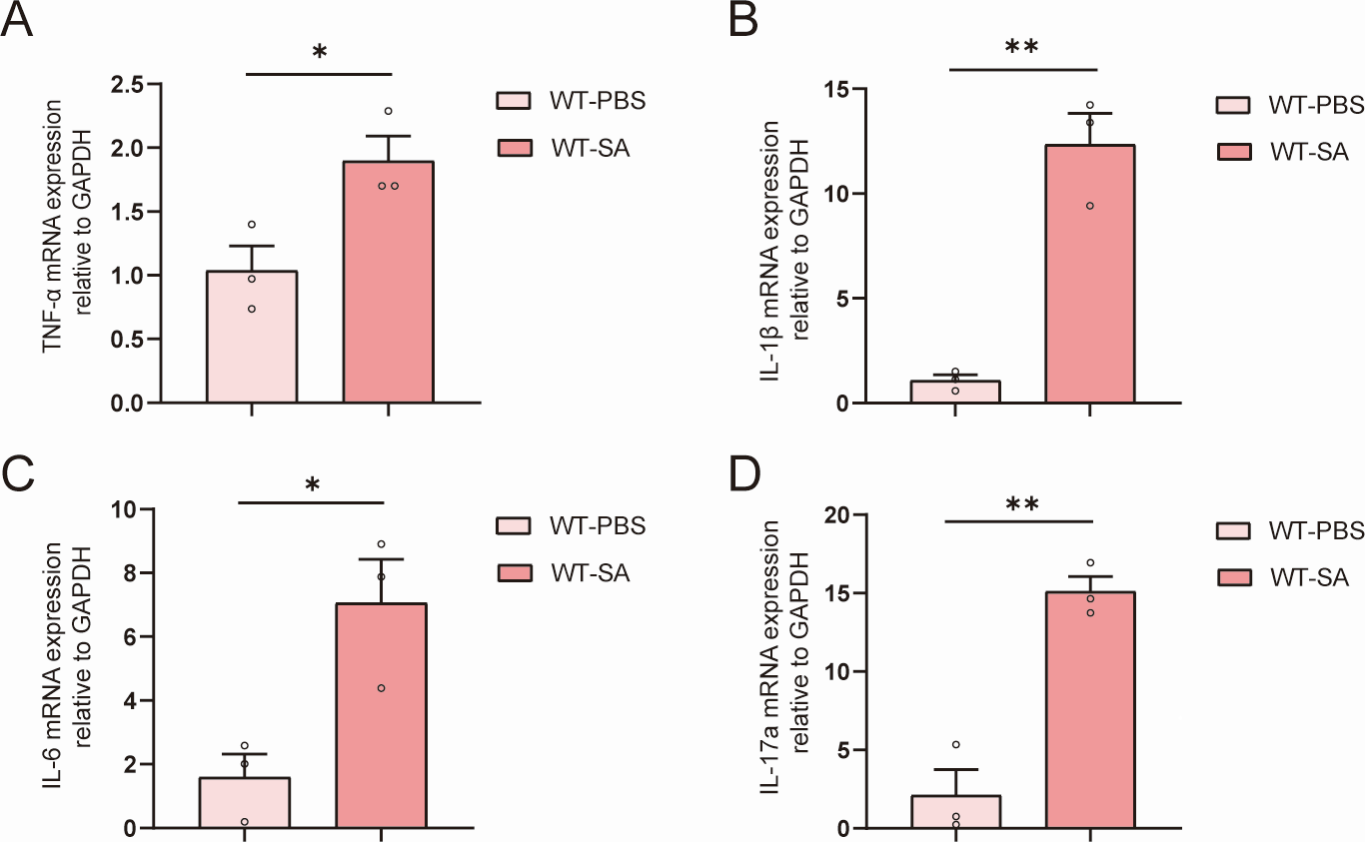
Host inflammatory cytokines are upregulated during *S. aureus* infection. (**A–D**) TNF-α, IL-1β, IL-6, and IL-17a mRNA expression levels were determined by real-time qPCR experiments (*n* = 3 mice for each group; for TNF-α, *P* = 0.0355; for IL-1β, *P* = 0.0017; for IL-6, *P* = 0.0242; and for IL-17a, *P* = 0.0023). All data are presented as the mean ± SEM. unpaired *t*-test was used. *P < 0.05, **P < 0.01, vs WT-PBS group.

Previous studies have reported that CNS produces inflammatory cytokines such as tumor necrosis factor alpha (TNF-α) and interleukin-6 (IL-6) after viral or bacterial invasion (28–30). In this study, we investigated the changes of host inflammatory cytokines in the *S. aureus-*induced brain abscess model mice using real-time qPCR and found that the expression of inflammatory cytokines such as TNF-α, IL-1β, IL-6, and IL-17a was increased significantly compared to that of the phosphate-buffered saline (PBS) control group (Fig. 2). These results indicate that *S. aureus* infection not only increases complement C3 levels but also activates inflammatory pathways in *S. aureus-*induced brain abscess.

**Fig 3:**
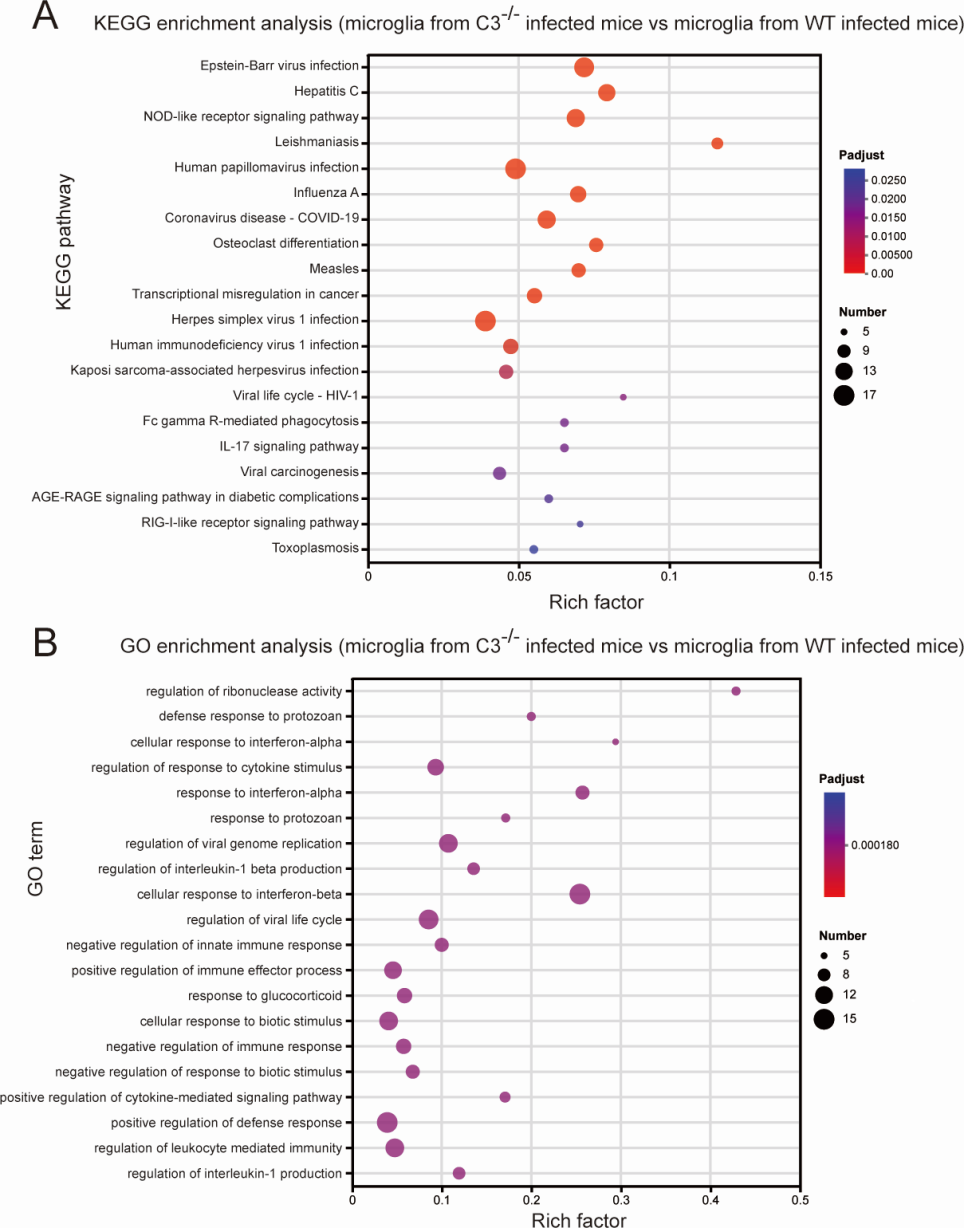
C3 mediates the inflammatory response of microglia. (**A**) KEGG enrichment analysis of RNA sequencing results of microglia from C3^−/−^ infected mice vs microglia from WT infected mice. (**B**) GO enrichment analysis of RNA sequencing results of microglia from C3^−/−^ infected mice vs microglia from WT infected mice.

To further elucidate the relationship between pro-inflammatory cytokines and C3, *S. aureus-*induced brain abscess modes of C3^−/−^ mice and wild-type (WT) mice were prepared, and microglia from C3^−/−^ or WT brain abscess mice were isolated using magnetic beads for further RNA sequencing analysis. Through Kyoto Encyclopedia of Genes and Genomes (KEGG) pathway enrichment analyses, we found that inflammation-related pathways, such as the NOD-like receptor signaling pathway, Fc gamma R-mediated phagocytosis, IL-17 signaling pathway, and RIG-I-like receptor signaling pathway, were also enriched (Fig. 3A). This suggests that C3 can mediate the inflammatory response of microglia. The Gene Ontology (GO) enrichment analysis also revealed the enrichment of biological processes related to the regulation of inflammatory responses, such as “regulation of interleukin-1 beta production” (Fig. 3B). The enrichment of these processes further supports the critical role of C3 in the inflammatory response of microglia.

### Transcriptomic changes in *S. aureus* after invading CNS

**Fig 4:**
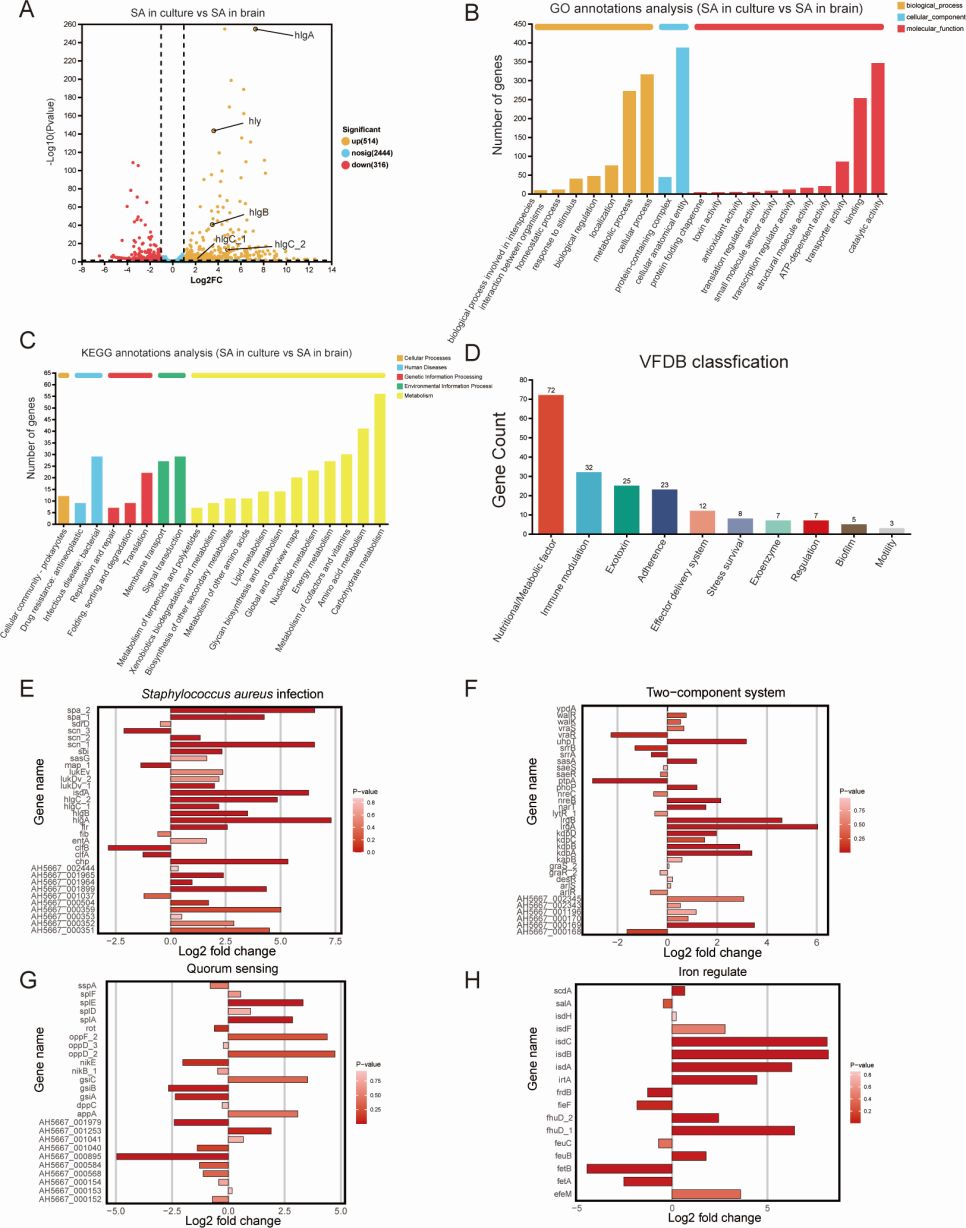
Transcriptomic changes in *S. aureus* after invading the host. (**A**) Volcano plot for genes (SA in culture vs SA in brain). (**B**) GO annotation analysis of the genes (SA in culture vs SA in brain), including biological process, cellular component, and molecular function. (**C**) KEGG annotation analysis of the genes (SA in culture vs SA in brain), including cellular processes, human diseases, genetic information processing, environmental information processing, and metabolism. (**D**) Virulence Factor Database (VFDB) classification of RNA sequencing results of SA in culture vs SA in brain. (**E**) Expressions of the genes associated with *S. aureus* infection of RNA sequencing results of SA in culture vs SA in brain. (**F**) Expressions of the genes associated with the two-component system of RNA sequencing results of SA in culture vs SA in brain. (**G**) Expressions of the genes associated with quorum sensing of RNA sequencing results of SA in culture vs SA in brain. (**H**) Expressions of the genes associated with iron regulate of RNA sequencing results of SA in culture vs SA in brain.

We hope to predict the changes in bacterial genes when *S. aureus* infects the host. Therefore, we conducted RNA sequencing to compare transcriptional alteration of *S. aureus* in the culture medium and the striatum of infected mice. In the sequencing results, 514 genes were upregulated and 316 genes were downregulated in the transcriptomic changes comparing bacteria *in vivo* and *in vitro* (Fig. 4A). The sequencing results were subjected to a variety of analytical methods for interpretation.

In the GO annotation analysis, differentially expressed genes were classified into three categories: cellular component (CC), molecular function (MF), and biological process (BP). Among the three categories, genes were most primarily enriched in the cellular anatomical entity (Fig. 4B). Our results showed that genes associated with the CC pathway exhibit fewer changes compared to those associated with the MF and BP pathways. It was suggested that during bacterial invasion of CNS, *S. aureus* undergoes more significant changes in biological processes such as regulatory signaling and metabolic processes. Through KEGG annotation analysis (Fig. 4C), we found that the differentially expressed genes were primarily enriched in metabolism-related pathways, which indicated that genes facilitating the identification and enhancement of pathogen metabolism are activated in significant quantities during the invasion of the host, thereby assisting in the complete invasion process.

Subsequently, an analysis of the bacterial virulence regulation was conducted by using Virulence Factor Database (31), and we found that virulence-related genes were mainly enriched in nutritional/metabolic factors, immune modulation, exotoxin, adherence, effector delivery system, and stress survival (Fig. 4D). In order to gain deeper insight into the precise alterations in virulence-related regulation that occur during pathogen infection, in addition to a comprehensive interpretation of the sequencing results, we conducted a detailed examination of some key mechanisms underlying virulence regulation. Initially, we conducted an enrichment analysis of the genes associated with *S. aureus* infection and found that the majority of genes involved in bacterial virulence exhibited elevated expression. Notably, we observed significant upregulation of hemolysin genes (*hlgA* [gamma-hemolysin component A], *hly* [alpha-hemolysin], *hlgB* [gamma-hemolysin component B], *hlgC_1* [gamma-hemolysin component C], and *hlgC_2* (gamma-hemolysin component C]) in the immune modulation, exotoxin, and stress survival pathways (Fig. 4E). The high level of expression of the hemolysin genes not only ranked high in the expression of genes associated with *S. aureus* infection but also stood out in all *S. aureus* gene comparisons (Fig. 4A). The results suggest that the hemolysin family genes play a key role in the process of *S. aureus* invasion into the CNS.

Except for the significant alterations observed in the hemolysin family, other related virulence genes of *S. aureus* also have notable expression changes. The genes related to the two-component regulatory system (TCS) were subjected to analysis (Fig. 4F), and the results indicated an overall increasing trend. TCS is a signaling pathway that enables the bacteria to sense and respond to environmental changes, including fluctuations in pH, temperature, and osmolarity (32–34). The elevated expression of TCS in pathogens amplifies the sensitivity of bacteria to external environmental fluctuations, augments the influx of external signals into the bacterial cytoplasm, and facilitates bacterial responsiveness to external killing measures. Furthermore, the quorum-sensing system (Fig. 4G) and the iron regulation system (Fig. 4H) were subjected to analysis. *S. aureus* monitors the quorum-sensing population density via signaling molecules known as auto-inducers, and this quorum-sensing system coordinates the expression of virulence factors, such as toxins and enzymes, allowing the bacteria to modulate its pathogenicity, depending on its numbers, ensuring that virulent behaviors are expressed at the optimal moment for infection progression (35–38). Iron regulation is another vital factor in *S. aureus* pathogenesis, as bacteria must acquire iron from the host as an essential nutrient (39). The regulation of iron uptake is tightly linked to both the TCS (40, 41) and quorum sensing (42), reflecting how these pathways interconnect to fine-tune bacterial adaptation during infection. In summary, our sequencing results indicate that *S. aureus* may enhance the survival, colonization, and invasion through regulating TCS, quorum sensing, iron, and so on, during bacterial invasion of CNS.

### Dual RNA-Seq reveals C3-mediated host inflammatory response

During infection, pathogens (such as viruses and bacteria) and host cells interact. resulting in significant changes in the gene expression of both (26, 27, 43). Although preliminary predictions have been made separately, which identified the genes and potential regulatory pathways involved in the changes in *S. aureus* and the host, the interaction between them remains unknown. To address this issue, we employed the dual RNA-seq technology, which can be used to simultaneously capture and analyze the gene expression changes of pathogens and host cells during the infection process. We used *S. aureus-*induced brain abscess modes of C3^−/−^ mice and WT mice for dual RNA-seq to reveal the impact of the complement molecule C3 on both the host and the bacteria.

**Fig 5:**
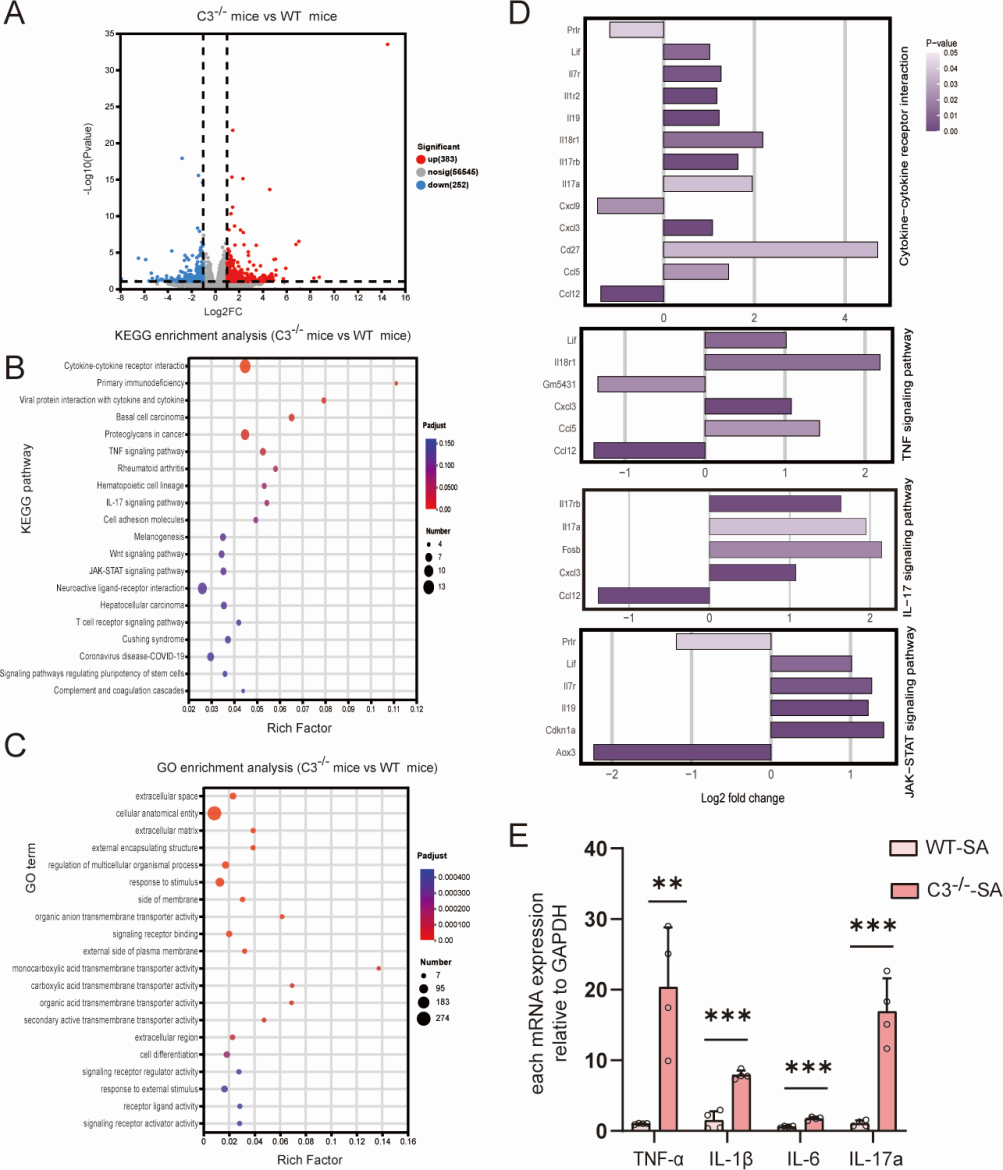
Dual RNA-seq transcriptomic changes in C3^−/−^ mice and WT mice. (**A**) Volcano plots for genes in C3^−/−^ mice vs WT mice. (**B**) KEGG enrichment analysis of the differential genes in C3^−/−^ mice vs WT mice. (**C**) GO enrichment analysis of the differential genes in C3^−/−^ mice vs WT mice. (**D**) Analysis of signaling pathway associated with inflammatory response in host with C3 deficiency. From top to bottom, each figure represents the following pathways: the cytokine-cytokine receptor interaction pathway, the TNF signaling pathway, the IL-17 signaling pathway, and the JAK-STAT signaling pathway. (**E**) Real-time qPCR analysis of changes in TNF-α, IL-1β, IL-6, and IL-17a during SA infection between C3^−/−^ mice and WT mice. **P < 0.01, ***P < 0.001.

In the host, we found 383 upregulated and 252 downregulated genes in the striatum of C3^−/−^ mice compared to WT mice after *S. aureus* infection (Fig. 5A). KEGG analysis revealed enrichment in multiple inflammation-related pathways, including cytokine-cytokine receptor interaction, primary immunodeficiency, tumor necrosis factor (TNF) signaling pathway, IL-17 signaling pathway, and Janus kinase (JAK)-signal transducer and activator of transcription (STAT) signaling pathway (Fig. 5B). Therefore, we speculated that the absence of C3 significantly impacts the host inflammatory response primarily by altering the gene expression of multiple key immune and inflammatory pathways and leads to abnormal inflammatory responses affecting the activity of several inflammatory signaling pathways during *S. aureus* infection. Moreover, the enrichment of cytokine-cytokine receptor interactions and TNF signaling pathways suggests alterations in host cytokine secretion and receptor-mediated signal transduction, potentially leading to an imbalance in inflammatory mediators, which may influence the recruitment and activation of immune cells. Additionally, the observed changes in the IL-17 and JAK-STAT signaling pathways underscore the significant impact of C3 deficiency on the regulation of the host immune response, particularly in modulating antibacterial immunity and promoting inflammatory processes.

GO analysis revealed changes related to response to stimulus and signaling receptor binding (Fig. 5C). These findings suggest that C3 deficiency may impair the ability of the host to recognize and respond to external pathogens, particularly during early-stage immune recognition and opsonization via the complement system. Furthermore, C3 deficiency alters pathways associated with stimulus response and signaling receptor binding, indicating that complement system dysregulation may not only compromise the host direct defense against *S. aureus* but also disrupt immune receptor-mediated signaling. This disruption could result in delayed or dysregulated immune responses, potentially exacerbating susceptibility to infection.

The deficiency of C3 has broad and profound effects on the host inflammatory response, especially by regulating and influencing the cytokine network and immune functions through several key signaling pathways (44, 45). To provide a more detailed explanation of the host immune response in the *S. aureus-*induced brain abscess, we conducted a more rigorous analysis and discussion of the host immune-related regulatory pathways in the results of dual RNA sequencing.

First, through the cytokine-cytokine receptor interaction pathway (Fig. 5D), C3 participates in the regulation of the complement system, which controls cytokine production and function. Second, through the TNF signaling pathway (Fig. 5D), C3 is closely related to the TNF signaling pathway. TNF-α is a key pro-inflammatory cytokine that activates inflammatory responses to combat pathogens (28). Our results indicated that the absence of C3 affects the responses of the TNF signaling pathway. Additionally, as for the IL-17 signaling pathway (Fig. 5D), the IL-17 signaling pathway is another important pro-inflammatory pathway that mainly recruits neutrophils and other immune cells to fight against foreign pathogens (46, 47). C3 enhances the inflammatory response in this process, and its absence will lead to an increase in IL-17 pathway activity. Moreover, C3 deficiency also affected the JAK-STAT signaling pathway (Fig. 5D), indicating that C3 might play a broader role in the coordination of immune functions. To further validate the changes in cytokine levels and confirm the involvement of key immune-related pathways, we performed RT-qPCR to quantify the expression of inflammatory cytokines IL-1β, TNF-α, IL-6, and IL-17a in *S. aureus-*induced brain abscess modes of C3^−/-−^ mice and WT mice. As shown in Fig. 5E, our results demonstrated a significant upregulation in the expression of these cytokines in C3-deficient mice compared to the WT controls, confirming the regulatory role of C3 in modulating these critical inflammatory markers.

### Dual RNA-Seq reveals host-derived C3 regulatory genes in *S. aureus*

**Fig 6:**
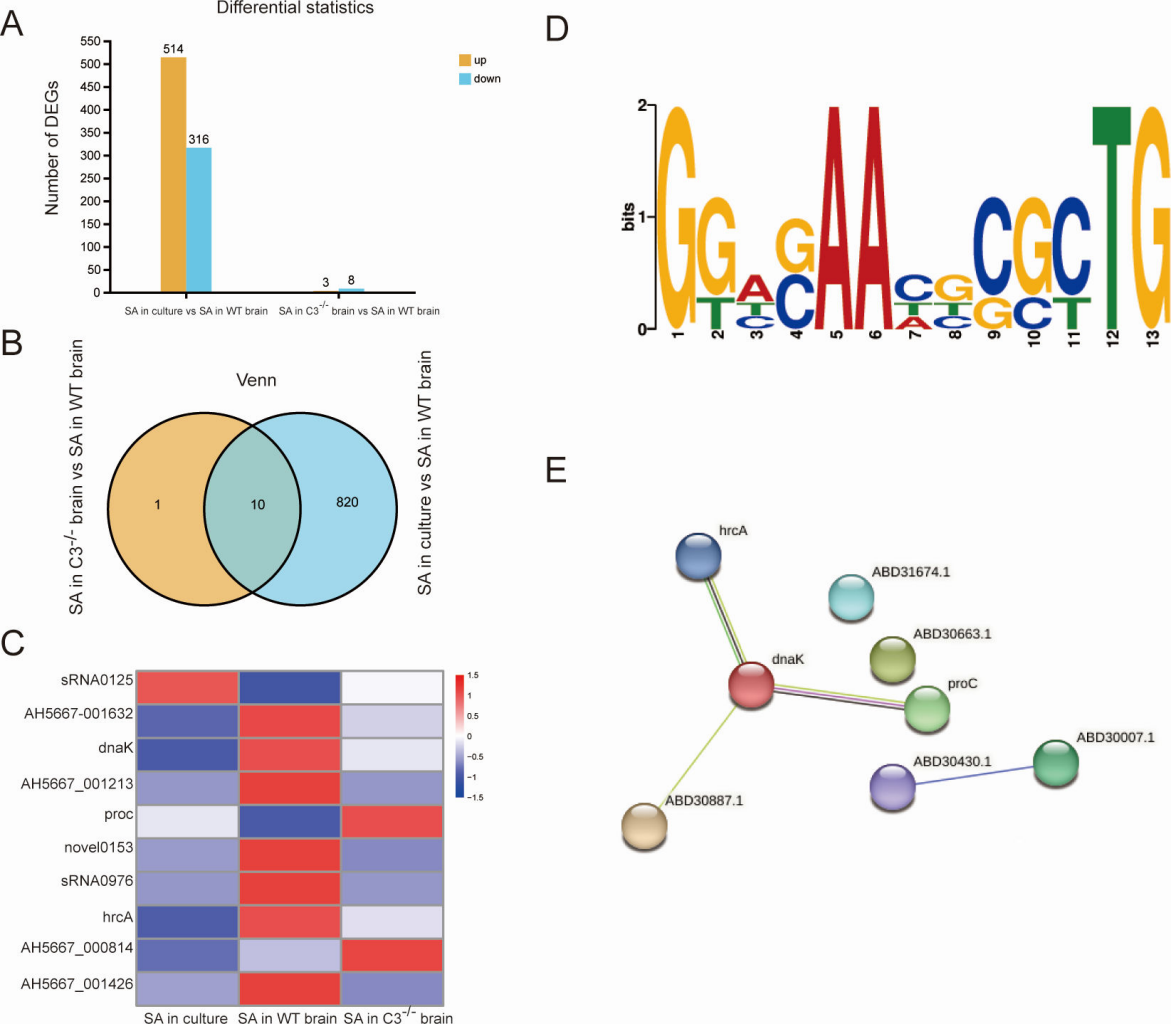
Host-derived C3 regulates genes related to *S. aureus*. (**A**) Differential statistics of number of differentially expressed genes (DEGs) between two sets of data (SA in culture vs SA in WT brain, SA in C3^−/−^ brain vs SA in WT brain). (**B**) Venn of number of DEGs of two sets of data (SA in culture vs SA in WT brain, SA in C3^−/−^ brain vs SA in WT brain). (**C**) Heatmap of DEGs of two sets of data (SA in culture vs SA in WT brain, SA in C3^−/−^ brain vs SA in WT brain). (**D**) Multiple Em for Motif Elicitation (MEME) analysis indicating the putative DNA-binding sequence for HrcA to be GGWSAAHBCGCTG, with lowest *P* value (3.74e-9). (**E**) Networks of interacting genes identified using Search Tool for the Retrieval of Interacting Genes/Proteins (STRING) from the intersection of the two sets of data (SA in culture vs SA in WT brain, SA in C3^−/−^ brain vs SA in WT brain). Nodes represent proteins. Edges represent interactions between proteins. Different colors represent different pathways and proteins.

It would be beneficial to gain further insight into the regulatory pathways and genes affected by C3 when *S. aureus* infects the host. Subsequently, an in-depth examination was conducted on the consolidated sequencing outcomes of the dual RNA-seq and *S. aureus*-related RNA-seq. We found that 514 genes were upregulated and 316 genes were downregulated after *S. aureus* infection, while 3 genes were upregulated and 8 genes were downregulated after C3 deficiency (Fig. 6A). We took the intersection of the two sets of data to obtain the genes that responded to C3 after *S. aureus* invasion of the host (Fig. 6B). We found a total of 10 genes in the intersection set, namely, sRNA0125, AH5667_001632, *dnaK*, AH5667_001213, *proC*, novel0153, sRNA0976, *hrcA*, AH5667_000814, and AH5667_001426 as shown in Fig. 6C.

HrcA acts as a negative regulator by interacting with the promoter regions of class I heat shock gene operons, thereby inhibiting the transcription of heat shock protein (48). We speculated that these 10 genes have mutual regulation. The first speculation was that HrcA acted as a transcription factor to regulate other genes. The results of the MEME (49) suite analysis indicated the putative DNA-binding sequence for HrcA to be GGWSAAHBCGCTG, with the lowest *P* value (3.74e-9) (Fig. 6D). The promoter region of *proC* has a potential binding region for HrcA, which was predicted by FIMO (50). This result suggests that HcrA may directly regulate the *proC* gene in *S. aureus*, which is constant with the previous report in *Mycobacterium tuberculosis* (48). Additionally, interactions exist between proteins. We used STRING to predict the interactions between proteins, and results indicated that these proteins form a regulatory network that revolves around *dnaK* as the core member (Fig. 6E). The interactions between HrcA and DnaK have been reported to mediate essential functions, including stress response, gene regulation, and biofilm formation, in species such as *Mycobacterium tuberculosis*, *Streptococcus pneumoniae*, and *Listeria monocytogenes* (51–54). The networks are shown schematically in Fig. 6E. Therefore, protein interactions form a tightly regulated network. We have preliminarily mapped the potential mechanisms by which *S. aureus* responds to host complement molecule C3.

### Δ*hla S. aureus* causes downregulation of inflammatory cytokines in brain abscess

**Fig 7:**
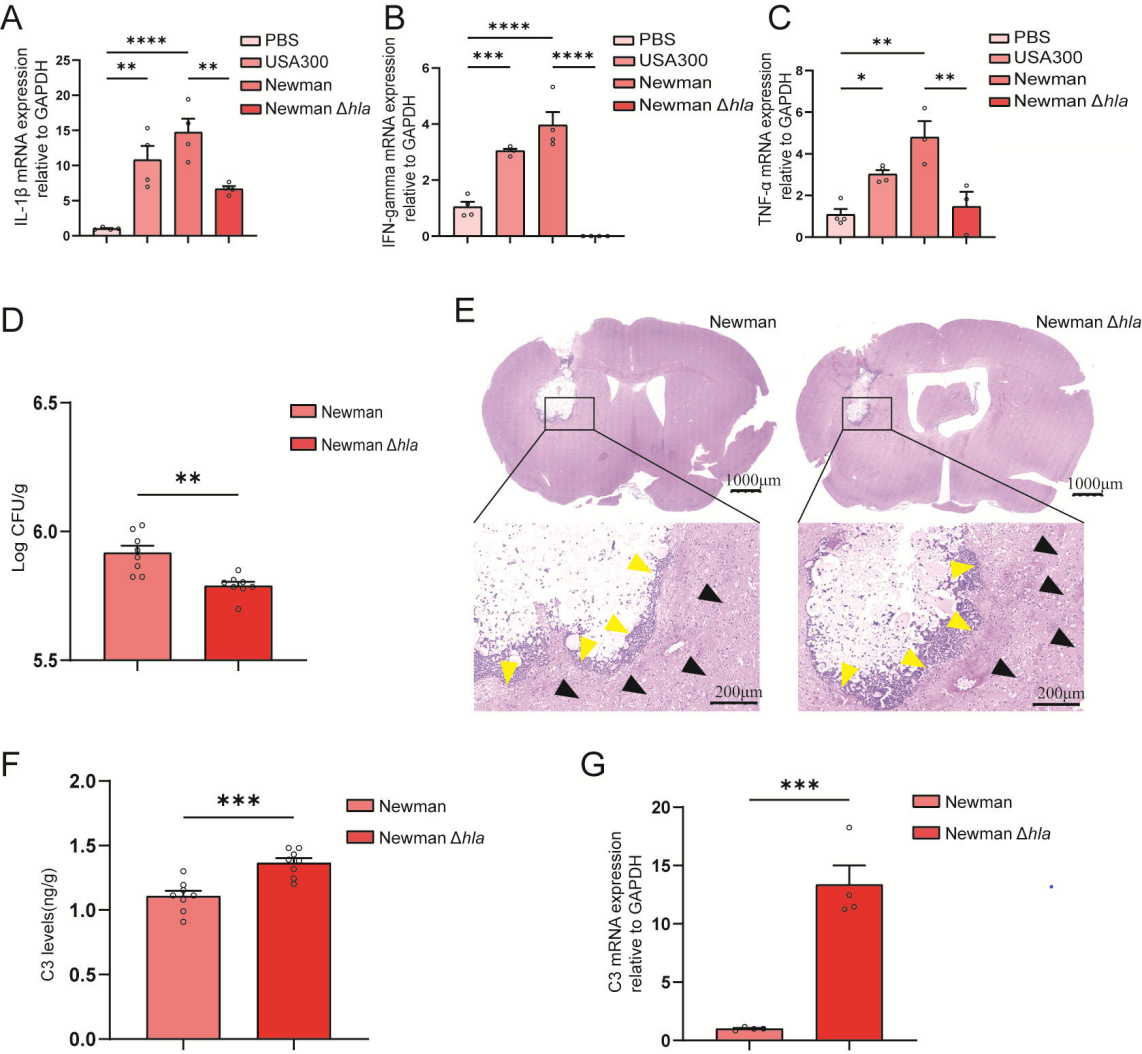
Δ*hla* of *S. aureus* causes downregulation of inflammatory cytokines after host invasion. (**A–C**) IL-1β, IFN-γ, and TNF-α mRNA expression levels were determined by real-time qPCR. (*n* = 3–4 for each group). All data are presented as the mean ± SEM. One-way analysis of variance with Bonferroni post hoc test was used. *P < 0.05, **P < 0.01, ***P < 0.001, ****P < 0.0001, vs PBS group. (**D–G**) The changes in the host following *S. aureus* infection in Newman vs Newman Δ*hla* are shown. All data are presented as the mean ± SEM. An unpaired *t*-test was used. **P  <  0.01, ***P  <  0.001, vs the Newman group. (**D**) Bacterial load in the brain (*n* = 8). (**E**) H&E staining results of the brain. (**F**) C3 expression detected by ELISA (*n* = 8). (**G**) C3 expression detected by real-time qPCR (*n* = 4).

To elucidate the roles of *hla* in *S. aureus-induced* brain abscess, we investigated the differences in host inflammatory responses to the alpha-hemolysin-deficient strain (Newman Δ*hla*) and WT strain of *S. aureus* by using the model mice of brain abscess. Real-time qPCR results indicated that compared to the WT strain, the Δ*hla* strain led to a downregulation of inflammatory cytokines such as TNF-α, IL-1β, and interferon gamma (IFN-γ) in BV2 cells (Fig. 7A through C). This indicates that alpha-hemolysin plays an important role in activating the pro-inflammatory response of microglia. In addition, bacterial load experiments revealed that the Δ*hla* strain had a significantly lower bacterial burden compared to the WT strain (Fig. 7D). Histological examination by hematoxylin and eosin (H&E) staining showed that the abscess area in the Δ*hla* strain was also smaller than that of the WT strain group (Fig. 7E), suggesting that the Δ*hla* strain induced less tissue damage. Moreover, we observed that the expression level of C3 was significantly lower following the Δ*hla* strain (enzyme-linked immunosorbent assay [ELISA] and RT-qPCR experiments, respectively, present in Fig. 7F and G), indicating that alpha-hemolysin may also be involved in modulating the complement system. Therefore, alpha-hemolysin may be a potential therapeutic target, and inhibiting its activity could reduce inflammation-related damage in the CNS.

## DISCUSSION

In this study, we investigated the bidirectional effects of *S. aureus* infection on host immune responses and the pathogen transcriptomic alterations, focusing specifically on the role of complement component C3. Our findings emphasize the pivotal role of C3 in regulating the host inflammatory response and modulating bacterial gene expression, further complementing and expanding upon prior research on the interactions between host immune factors and bacterial adaptation mechanisms (55, 56).

Consistent with previous research (28), our data confirmed that C3 plays a central role in amplifying the host inflammatory response to *S. aureus* infection. The observed upregulation of TNF-α, IL-1β, and IL-6 indicates that C3 is essential for activating key inflammatory pathways. When we knocked out C3, we found that inflammatory cytokines such as TNF-α, IL-1β, IL-17A, and IL-6 were upregulated on day 1 post-infection. This early cytokine surge suggests that in the absence of C3, the immune system attempts to compensate for the loss of complement activity by increasing the production of pro-inflammatory mediators (57).

Recent studies have also indicated that C3 is involved in modulating the activity of the JAK-STAT (58) and NOD-like receptor pathways (59), which are essential for both pathogen recognition and cytokine signaling. Our findings expand on these results by showing that C3 deficiency disrupts these pathways, leading to a suboptimal immune response that may contribute to increased susceptibility to bacterial infections. In addition, we also found that C3 deficiency has other impacts on the host. For example, we enriched the cytokine-cytokine receptor interaction pathway, which suggests that C3 may be involved in the imbalance of host inflammatory mediators. Furthermore, our results also indicate the presence of complement system dysregulation, which significantly impairs the host’s defense against pathogens and signaling pathways related to immune responses within the host.

Our transcriptomic analysis reveals significant changes in the gene expression profile of *S. aureus* during infection, with over 800 genes showing differential expression compared to *in vitro* conditions. There were several changes in bacterial virulence regulatory pathways, such as TCS, quorum sensing, and iron regulation. After bacterial entry into the host, the upregulation of most genes in these pathways serves as the main driving force for bacterial invasion of the host. Additionally, the upregulation of *hlgA*, *hly*, and *hlgC* aligns with findings by Tian et al. and Putra et al. (60, 61), highlighting the roles of alpha-hemolysin in corneal wound healing impairment, bacterial invasion, and immune evasion. This study elucidates the intricate interactions between *S. aureus* and the host during brain infection, uncovering a series of processes induced by the pathogen that are conducive to the survival within the host.

The differences in host inflammatory responses between Newman Δ*hla* and WT strain provided further evidence of the role of hemolysin in modulating host immune responses. The significant reduction in TNF-α and IL-1β levels in microglia infected with Δ*hla* indicates that alpha-hemolysin is a major driver of the pro-inflammatory response in the CNS. The results from bacterial load and H&E staining, consistent with the reports published by Zheng et al. (62), further support this finding. The Δ*hla* strain showed a lower bacterial load and a smaller abscess area, indicating reduced tissue damage and a less severe inflammatory response. These findings suggest that alpha-hemolysin not only triggers inflammatory cytokine production but also contributes to the persistence and spread of infection in the host.

One of the novel aspects of our study was the identification of C3-responsive genes in *S. aureus*, suggesting that host-derived C3 not only modulates immune responses but also directly influences bacterial gene expression. The identified 10 genes, including *proC* and *hrcA*, point to a complex regulatory network where C3 modulates bacterial metabolic and stress response pathways. The observation that *proC*, involved in amino acid biosynthesis, may be regulated by the stress response regulator HrcA aligns with findings that bacterial chaperones and proteases play crucial roles in stress adaptation and the regulation of virulence gene expression (48, 51, 63). Our analysis expands on this concept by linking C3 to the regulation of bacterial metabolic pathways, suggesting that the immune environment within the host directly impacts bacterial fitness and virulence.

The role of C3 in regulating the inflammatory response of microglia, the primary immune cells of the CNS, was evident from RNA-seq results. The enrichment of pathways like NOD-like receptor signaling and Fc gamma R-mediated phagocytosis in C3^−/−^ microglia aligns with recent studies showing the C3-C3aR pathway as a key mediator of microglial activation, neuroinflammation, and interactions with astrocytes, contributing to neurodegeneration and depression (24, 64, 65). Our results suggest that the absence of C3 leads to a reduced microglial response, potentially impairing the clearance of *S. aureus* from the CNS.

Our findings build upon and extend the current understanding of complement-pathogen interactions during bacterial infections. The impact of C3 on both host and pathogen responses underscores its multifaceted role in immune regulation. Future studies should focus on elucidating the precise molecular mechanisms underlying C3-mediated transcriptional changes in *S. aureus*, as well as exploring the therapeutic potential of targeting complement pathways in bacterial CNS infections. Additionally, investigating the role of C3 in other bacterial and viral infections could provide valuable insights into whether similar immune-modulatory effects are observed across diverse pathogens.

In conclusion, our study highlights the dual role of C3 in regulating both host and bacterial responses during *S. aureus* infection. The absence of C3 disrupts key inflammatory pathways, impairs pathogen recognition, and influences bacterial transcriptomic adaptation. These findings suggest that targeting complement pathways and bacterial virulence factors, such as alpha-hemolysin, could provide novel therapeutic strategies to combat CNS infections and reduce inflammation-induced tissue damage.

## MATERIALS AND METHODS

### Bacterial strains and cultures

The *S. aureus* strain (USA300) was carefully preserved at the highly regarded laboratory of the Second Affiliated Hospital of Soochow University. The *S. aureus* strains (Newman and Newman Δ*hla*) were graciously provided by Professor Xiancai Rao from the Army Medical University. The bacterial strains were initially cultured in tryptic soy broth (TSB) at 37°C with continuous shaking at 200 rpm overnight, and then a 1:100 dilution in TSB was prepared for further propagation.

### Cell lines and cultures

The BV2 microglial cell line, originally sourced from the Institute of Neuroscience at Soochow University, was maintained in high-glucose Dulbecco's modified Eagle medium (HyClone, SH30243.01, USA) supplemented with 10% fetal bovine serum (Gibco, 16000–044). The cells were exposed to *S. aureus* at a multiplicity of infection of 5:1, with the invasion period set to 2 h.

### Animal experiments

We followed previously reported methods for the construction of brain infection models. First, we used low-melting point agarose to prepare agarose spheres containing *S. aureus*. The control group was replaced with PBS. Five microliters of encapsulated *S. aureus* agarose bead suspension (1 × 10^5^ CFU) was carefully injected into the right striatum at the indicated coordinates: rostral, 0.8 mm; right bregma, 2 mm; and ventral bregma, 3.0 mm. Controls were injected with agarose spheres and replaced with PBS. The needle was held in the original position for 5 minutes. The needle was removed; the skin was carefully sutured; and the mice were returned to their cages.

### RNA extraction, library construction, and sequencing

#### Sequencing process for prokaryotes

Total RNA was extracted from tissue samples, and the concentration and purity of the extracted RNA were examined using NanoDrop 2000; RNA integrity was detected by agarose gel electrophoresis, and RNA integrity number (RIN) values were determined by Agilent 2100. Removal of rRNA and addition of fragmentation buffer can randomly break the mRNA into small fragments of about 200 bp. Under the action of reverse transcriptase, one-stranded cDNA was synthesized by reverse transcription using mRNA as a template with random primers. dUTP was used instead of dTTP in dNTP reagent for two-stranded synthesis, so that the bases in the second chain of cDNA contained A/U/C/G. The structure of double-stranded cDNA was sticky ended, which was patched up to a flat end by adding End-Repair Mix, followed by adding an A to the 3′ end. The double-stranded cDNA structure has a sticky end, which was made up of a flat end by adding End-Repair Mix, followed by the addition of an A base at the 3′ end for a Y-shaped connector. Prior to PCR amplification, the second strand of cDNA was digested with UNG enzyme so that only the first strand of cDNA was included in the library. Sequencing was performed on the NovaSeq X Plus platform.

#### Sequencing process for eukaryotes

Total RNA was extracted from the tissue samples, and the concentration and purity of the extracted RNA were examined using NanoDrop 2000. RNA integrity was examined by agarose gel electrophoresis, and RQN values were determined by Agilent 5300. The mRNA was isolated from the total RNA by A-T base pairing using magnetic beads with oligo(dT) and poly(A). mRNA could be randomly broken into small fragments of about 300 bp by adding a fragmentation buffer and choosing suitable conditions. Under the action of reverse transcriptase, using random primers, one-stranded cDNA is synthesized in reverse using mRNA as a template, followed by two-stranded synthesis to form a stable double-stranded structure. The double-stranded cDNA structure is sticky ends, which are patched to flat ends by adding End-Repair Mix, followed by an A base at the 3′ end to facilitate the later addition of adapter sequences. On-board sequencing was performed on the NovaSeq X Plus platform.

### Real-time qPCR

A reaction system was prepared and added to the 96-well plate. The system mixture included cDNA, forward primer, reverse primer, and fluorescent DNA-binding dye (Taq Pro Universal SYBR qPCR Master Mix, vazyme, Q712-02), and RNase-free water. The reaction conditions were set up, and the fluorescence signal was detected by a real-time PCR system. The levels of relative mRNA expression were analyzed using the 2^−∆∆Ct^ method. The following primers were used: C3 (forward primer: TTCTCCGCAGAGTTTGAGGT, reverse primer: TTCTTATCGCCATCCTGGAC); TNF-α (forward primer: CGGGCAGGTCTACTTTGGAG, reverse primer: ACCCTGAGCCATAATCCCCT); IL-1β (forward primer: CTTCAGGCAGGCAGTATCACTCAT, reverse primer: TCTAATGGGAACGTCACACACCAG); IL-6 (forward primer: GGGACTGATGCTGGTGACAA, reverse primer: TCTGCAAGTGCATCATCGTT), and IL-17a (forward primer: TCAGCGTGTCCAAACACTGAG, reverse primer: CGCCAAGGGAGTTAAAGACTT).

### Immunofluorescence staining and fluorescent microscopy

Mice were perfused with 4% paraformaldehyde (PFA). Brains were rapidly harvested, fixed in 4% PFA, and cryo-protected. Thirty micrometer coronal sections were cut with a microtome and were fixed in a refrigerator with negative 80°C on positively charged slides. When the slides were removed again for immunofluorescent staining, they were heated in an oven at 60°C and washed with PBS and PBS-Tween 20 (PBST). The primary antibodies for C3 (1:500; abcam, ab11862) and Iba-1 (1:500, Oasis Biofarm OB-PGP049) were used. The slides were blocked with PBST containing 5% goat serum for 1 h at room temperature, then incubated overnight at 4°C in primary antibody diluted in blocking solution. After being washed with PBST three times, the slides were incubated for 2 h in appropriate secondary antibodies. The second antibodies were goat anti-guinea pig Alexa Fluor 488 (Oasis Bioform; GP488, 1:500) and goat anti-rat IgG (H+L) Alexa Fluor 647 (Cell Signaling Technology; 4418, 1:500). All sections were stained with 4′,6-diamidino-2-phenylindole (Thermo Fisher Scientific; D1306, 1:500). The slides were mounted and coverslips were applied after final washing. Images were obtained using LSM900 laser-scanning confocal microscope (Carl Zeiss, Germany). Images were analyzed for fluorescence intensity and colocalization using ImageJ software.

### Western blot

The homogenization of the striatum region was effectively achieved using a lysis buffer containing phosphatase and protease inhibitors. Subsequently, the protein extracts were carefully loaded onto 12.5% polyacrylamide gels and subjected to initial electrophoresis at 80 V to concentrate the gel and delineate the gel boundary. This was followed by electrophoresis at 120 V to facilitate the effective separation of protein bands. Following this, the proteins were transferred to a polyvinylidene fluoride membrane, which was then blocked with 5% skim milk. The membrane was incubated overnight at 4°C with the specified primary antibodies: rabbit anti-IL-1β (Abclonal; A16288, 1:1,000) and mouse anti-β-actin (FUDE; FD0060, 1:1,000). After three washes of 15 minutes each with Tris-buffered saline with Tween-20 (TBST), the membranes were incubated at room temperature for 2 h with the following secondary antibodies: goat anti-rabbit HRP (FUDE, FDR007) and goat anti-mouse HRP (FUDE, FDM007). Protein bands were visualized using an enhanced chemiluminescence detection kit (New Cell & Molecular Biotech, P10300). The intensity of each band was analyzed using ImageJ.

### ELISA

One day after intracranial inoculation of *S. aureus* WT or Δ*hla* strains, the mice were euthanized, and the striatum was collected. Nine times the tissue weight in volume of PBS was added (g:mL = 1:9), and the sample was homogenized thoroughly using a homogenizer. Centrifugation was performed at 2,500 rpm for approximately 20 minutes, and the supernatant was carefully collected. The instructions provided by the reagent kit (LV30768) were followed. Fifty microliters standard or sample was added to each well and incubated for 50 minutes at 37°C. The plates were washed three times. One hundred microliters biotinylated antibody working solution was added to each well and incubated for 50 minutes at 37°C. The plates were washed three times. One hundred microliters SABC Working Solution was added into each well and incubated for 30 minutes at 37°C. The plates were washed three times. One hundred microliters TMB Substrate Solution was added and incubated for 15 minutes at 37°C. Fifty microliters Stop Solution was added, read at 450 nm immediately, and calculated.

### Bacterial load counting in mouse brain

One day after intracranial inoculation of *S. aureus* WT or Δ*hla* strains, the mice were euthanized, and the striatum was collected. The tissue was homogenized in PBS with 1% Triton X-100. Then, a series of 10-fold dilutions were made on TSB agar plates for colony counting. The plates were incubated at 37°C for 24 h, and the number of colonies was counted.

### Histological examination

One day after intracranial inoculation of *S. aureus* WT or Δ*hla* strains, the mice were euthanized, and the brains were removed and fixed in 4% paraformaldehyde. The infiltrated brain tissue was then embedded in paraffin. Continuous 4 µm thick sections were taken from the entire brain and stained with H&E. Each section was examined and photographed under a light microscope at ×2 or ×40 objective magnification.

### Statistical analysis

Statistical analyses were conducted using GraphPad Prism version 8. Data were expressed as the mean ± SEM. An unpaired two-tailed Student’s *t*-test was performed to compare the two groups. In cases involving more than two groups, one-way or two-way analysis of variance was applied, followed by Tukey’s post hoc test to evaluate pairwise differences among the group means.

## Data Availability

The raw RNA-seq data is available by accession numbers PRJNA1182970 and PRJNA1205542 in the National Center for Biotechnology Information Sequence Read Archive database.
